# Representative sample survey on factors determining the Czech physicians’ awareness of generic drugs and substitution

**DOI:** 10.1186/s12913-019-4631-y

**Published:** 2019-10-30

**Authors:** J. Maly, E. Zimcikova, J. Babica, A. A. Kubena, J. Kostriba, K. Mala-Ladova

**Affiliations:** 1 Faculty of Pharmacy in Hradec Kralove, Department of Social and Clinical Pharmacy, Charles University, Akademika Heyrovskeho 1203, 500 05 Hradec Kralove, Czech Republic; 2 Faculty of Pharmacy in Hradec Kralove, Czech Pharmaceutical Museum, Charles University, Akademika Heyrovskeho 1203, 500 05 Hradec Kralove, Czech Republic

**Keywords:** Generic drugs, Generic substitution, Physician, Representative survey, Czech Republic

## Abstract

**Background:**

Generic drugs and generic substitution belong to the tools by which healthcare costs may be reduced. However, low awareness and reluctance among healthcare professionals towards generic drugs may negatively affect the rational use of generic substitution.

**Methods:**

The study aimed to analyze opinions and attitudes towards generic drugs and generic substitution among Czech physicians including their understanding of generic substitution legislative rules and the physicians´ previous experience in this field. Using random allocation, 1551 physicians practicing in the Czech Republic were asked to participate in the sociological representative survey conducted from November to December 2016, through face-to-face structured interviews comprising 19 items. Factor analysis as well as reliability analysis of items focused on legal rules in the context of physicians’ awareness were applied with *p*-value of < 0.05 as statistically significant.

**Results:**

Of a total of 1237 (79.8%) physicians (43.7% males; mean age 47.5 ± 11.6 years, 46.3% general practitioners) 24.8% considered generic drugs to be less safe, especially those with specialized qualification (*p* < 0.01). However, only 4.4% of the physicians noticed any drug-related problems, including adverse drug reactions associated with generic substitution. The majority of physicians felt neutrally about performing generic substitution in pharmacies, nor they expressed any opinion on characteristics of generics, even though a better understanding of the legislation and higher need of accordance of substituted drugs were associated with more positive attitudes towards generic substitution (*p* < 0.05). Physicians showed low knowledge score of legislative rules (mean 3.9 ± 1.6 from maximum 9), nevertheless they overestimated the law, as they considered some rules valid, even if the law does not require them. Cronbach alpha of all legislative rules that regulate generic substitution increased from 0.318 to 0.553 if two optional rules (physician consent and strength equivalence) would be taken into account.

**Conclusions:**

There is no sufficient awareness of generic drugs and generic substitution related issues among Czech physicians, although a deeper knowledge of legislation improves their perception about providing generic substitution.

## Background

Generic drugs are off-patent products containing the same active substances as the previously approved brand name drug with the same bioequivalence, the same dosage form, the same route of administration, and the same therapeutic characteristics [1]. According to the European Medicines Agency, drug bioequivalence is defined as the ratio of pharmacokinetic parameters (maximum plasma concentration, C_max_, and area under curve, AUC) ranging from 80 to 125% (90% confidence interval). Two drugs are assumed to be therapeutically equivalent if they are claimed bioequivalent [2, 3].

One of the reasons for introducing generics to the market was to reduce healthcare costs [4, 5], not only because the development of generic drugs is cheaper, but also because pharmaceutical companies are competing for their market place [6]. It gives space that, for example, pharmacists can substitute brand name drugs for less expensive generic alternatives. Moreover, since the introduction of generic drugs brought better access to medicines and healthcare for patients in general, cost savings could be redirected to the area of rare or costly diseases [7, 8].

Generic drugs have been available in both acute and chronic disease therapies for many years. In 2013, the proportion of prescriptions filled with generic drugs ranged from 17% in Switzerland to 83% in the United Kingdom [9]. In the same year, generic drugs accounted for almost 40% in the Czech Republic (CR), while the increasing or sustained trend in prescribing generic drugs is still apparent in other countries as well [10]. Generally, despite the increased acceptance of generics, some mistrust and lack of confidence among stakeholders still prevail [11]. Previously published literature have shown that negative opinions about generic drugs in terms of effectiveness, quality, or safety are apparent both in the group of health professionals – physicians and pharmacists [3, 12, 13] – and in the lay public [14] across different geographical areas. There are several studies assessing patients´ attitudes, however, the opinions of prescribing physicians are also crucial since it often reflects patients’ behavior. Managing mutual partnerships between healthcare providers and patients can therefore decisively influence the use of generic drugs and generic substitution [11]. According to a recent systematic review, physician-related factors belong to the seven domains that play a significant role in the implementation and sustainability of generic substitution in healthcare. Consequently, the physicians´ knowledge is essential in establishing future policies, education, as well as interventions supporting accurate generic drug use in clinical practice [15].

In the CR, GS was legalized in December 2007 [16]. Pharmacists may substitute the prescribed drug for its alternative upon the patient’s consent, also after considering all the possible generic substitution related risks especially so as to reduce the patient’s financial burden. In short, drugs are regulated by price (maximum price) and reimbursement in the CR. The maximum price is determined based on the external and internal price referencing. External approach embraces an average of the three lowest prices from reference countries. If not applicable, the maximum ex-factory price is determined based on the price of the closest therapeutically comparable drug in the CR or in the reference basket countries. Reimbursement is always determined identically for all interchangeable drugs that are listed in the reference group. These drugs have similar efficacy, safety, and position in clinical practice. Reimbursement price is set according to the lowest price of drug within a reference group in the European Union, while all drugs within the same reference group have the same reimbursement price for the usual daily therapeutic dose. Currently, the price and reimbursement of the first generic drug has to be at least 40% lower than the price and reimbursement of the reference drug in the CR. In the case of another generic drug, the only the price is decreased [17, 18].

The first studies in the group of Czech general practitioners (GPs) and pharmacists, conducted a year after the legislative move in 2007, reflected distrust on account of low awareness of principles and results of bioequivalence, also due to inadequate knowledge of legislation, as well as based on negative personal experience [3, 19]. Nevertheless, pharmacists apparently perceived generic drug aspects more appropriately [20]. A current study follows up a previous survey (realized in 2008–2009), on the GPs´ views and attitudes towards generic substitution. The aim was to analyze opinions and attitudes towards generic drugs and generic substitution among a representative sample of Czech physicians including their understanding of legislative requirements for providing generic substitution, also inquiring the physicians´ experience in this field, almost a decade after the introduction of generic substitution in the CR.

## Methods

### Participants and setting

The sociological cross-sectional survey was conducted during November and December 2016 through face-to-face structured interviews. Such surveys have been performed regularly since 1995, in which trained interviewers asked both lay people and health professionals on different healthcare issues in the CR, including their opinions on and the level of awareness of the development of health services, prevention, or therapeutic strategies. In our survey, the Czech physicians´ opinions and attitudes towards generic drugs and generic substitution were explored by structured questions. The local Ethical Committee determined that the current study did not need formal ethical approval in conformity with national regulations. The study was conducted according to the principles stipulated by the Declaration of Helsinki and ICC/ESOMAR International Codex on Market, Opinion and Social Research and Data Analytics [21].

A national sample survey, in which equal representation of gender, age characteristics, as well as regional distribution of clinical practice was ensured by random allocation of 1551 physicians practicing in the CR who were asked to participate in the study. Participation was anonymous and voluntary as well as participants were fully aware of the purpose, nature, potential benefits or risks of the study. There were GPs for adults, GPs for children and adolescents and other medical specialists except dentists. Parameters for addressing the convenient participants came from the Institute of Health Information and Statistics by Ministry of Health database [22].

Except for data on socio-demographic characteristics (3 items) and medical specialties (3 items), the survey (Additional file 1) focused on the opinions on 10 statements related to brand name drugs, generic drugs and generic substitution. Responses were recorded on a five-point Likert scale (from strongly agree to strongly disagree). Further, previous experience with drug-related problems of generic drugs and providing generic substitution to patients (1 item), understanding of 9 legal rules for generic substitution in the CR (1 item with multiple choice), as well as attitudes towards performing generic substitution in pharmacies (1 item on Likert scale from positive to negative) were also solicited. The questionnaire was developed at the Department of Social and Clinical Pharmacy, Faculty of Pharmacy in Hradec Kralove, Charles University, according to the published material explained in detail elsewhere [19]. A pilot test was performed with 156 responding physicians to reveal the understanding of the questions and comprehensibility of the survey. Finally, the understanding of legal rules and attitudes towards generic substitution of GPs for adult patients were compared with previously published results [19].

### Statistical analysis

For the characteristics of the tested cohort, descriptive statistics was expressed as either absolute and relative frequencies or metric items given as the median, lower (25%) and upper (75%) quartiles (IQR), or mean ± standard deviation (SD). Pearson Chi-Square test was processed for correlation analysis by SPSS, version 20.0. Reliability analysis (Cronbach alpha) of items focused on legal rules as well as factor analysis for reduction of legal rules to independent factors were also applied [23, 24]. Kendall tau (τ) correlation and Kruskal Wallis test as appropriate in attitudes analysis as well as plot generation were performed using Wolfram, Mathematica, version 11.2 (Wolfram Research Inc.). Indeed, t-test was used for comparison between current and previously published results of GP cohort. A *p*-value of < 0.05 was considered statistically significant. In addition to the statistical significance value, the corresponding effect size was calculated based on Cohen’s convention (small-medium-large) [25].

## Results

### Characteristics of participants

A total number of 1237 (79.8%) respondents agreed to participate, of which 540 (43.7%) were male, mean age 47.5 ± 11.6 years (median 48; IQR: 38–58), and 255 (20.6%) respondents reported the capital city, Prague, as their place of practice. Gender, age categories, and locations of clinical practice were distributed with deviation of 0.1, 0.3 and 0.1% of the general sample in the CR, respectively. Physicians who refused to be involved in the survey (304; 20.2%) mainly reported lack of time (61.2%), no interest (22.4%), or distrust in any such research (6.9%).

There were GPs for adults (352; 28.5%), GPs for children and adolescents (220; 17.8%), obstetricians and gynecologists (172; 13.9%) and other medical specialists (493; 39.8%), especially internists and surgeons. Approximately half of the respondents (642; 51.9%) were working in the private sector institution. Most of the respondents had completed specialized qualification (1028; 83.1%), more significantly in older, or practicing in the private sector (*p* < 0.001).

### Opinions on brand name drugs, generic drugs and generic substitution

Physicians’ opinions on statements related to generic and brand name drugs as well as generic substitution are summarized in Table 1. Concerning the therapeutic equivalence between generic and brand name drugs, there were mostly positive opinions among the respondents (749; 60.6%), more frequently in GPs for children and adolescents (*p* < 0.001). Indeed, positive opinions outweighed in number, especially in male sample (*p* < 0.05), the view that generic drugs are therapeutically equivalent to each other (697; 56.3%). A slightly lower agreement was reported in terms of bioequivalence. Generic drugs in respect to brand name drugs were considered to be bioequivalent mostly by physicians in ambulatory care, more often than by physicians working in inpatient settings (p < 0.05). Interestingly, about one third of respondents (364; 29.4%) expressed no opinion at all on bioequivalence, as well as almost one quarter of respondents (276; 22.3%) did not even know whether the results of bioequivalence may be useful for their responsible decision-making process.

**Table 1:** Opinions on statements related to brand name drugs, generic drugs, generic substitution (*N* = 1237)

Statement	Strongly agree	Agree	Neutral	Disagree	Strongly disagree	Mean	SD
Every generic drug is therapeutically equivalent to the brand name drug.	15.8%	44.8%	20.5%	16.7%	2.2%	2.4	1.014
Every generic drug is therapeutically equivalent to any other generic drug.	10.7%	45.6%	25.5%	16.4%	1.8%	2.5	0.947
Every generic drug is bioequivalent to the respective brand name drug.	12.0%	41.5%	29.4%	14.9%	2.2%	2.5	0.957
I need more information on results of bioequivalence studies to make a responsible decision on the use of generic drugs.	31.0%	36.5%	22.3%	9.1%	1.1%	2.1	0.991
Every generic drug is of lower quality than the brand name drug.	6.3%	23.4%	27.7%	35.3%	7.3%	3.1	1.054
Every generic drug is less effective than the brand name drug.	5.3%	19.5%	26.4%	39.9%	8.9%	3.3	1.041
Every generic drug cause more adverse drug reactions than the brand name drug.	5.4%	23.1%	31.0%	33.7%	6.8%	3.1	1.019
Every generic drug is less costly than the brand name drug.	19.5%	42.4%	23.0%	12.9%	2.2%	2.4	1.004
The law imposes the same safety requirements on both generic and brand name drugs.	37.5%	36.8%	18.8%	5.7%	1.2%	2.0	0.949
Generic substitution reduces drug costs in the patient’s pharmacotherapy.	21.4%	42.0%	27.1%	7.9%	1.6%	2.3	0.937

*1 strongly agree, 2 agree, 3 neutral, 4 disagree, 5 strongly disagree*

*SD standard deviation*

Generic drugs were regarded as equal to brand name drugs in terms of quality, effectiveness, and incidence of adverse drug reactions (ADRs) by most of the physicians. However, a relatively large proportion of respondents were not able to express any opinion. Concerning ADRs, no opinion was reported particularly among physicians preparing for specialized qualification, opposed to the physicians who passed qualification, as they considered generic drugs to be less safe (*p* < 0.01). On the other hand, 1183 (95.6%) of respondents have noticed no ADRs or other drug-related problems associated with generic substitution in their patients in the previous 3 months. If any negative reactions at all, allergic reactions, ineffectiveness and highlighted ADRs of generic drugs were reported most commonly, particularly by physicians aged 40–59 years (*p* < 0.05).

### Understanding the legislation for providing generic substitution in pharmacies

Apart from items based on incorrect answers (such as false assumptions on the requirement of physician’s consent and strength equivalence), all the 7 legal requirements of providing generic substitution in pharmacies must be respected, according to the Czech legislation in force (Table 2). Each correct response scored one point (of maximum nine points) and the respondents gave correct answers to 3.9 ± 1.6 questions on average (median 4; IQR: 3–5). Respondents mentioned both physician’s consent and same strength as correct answers quite often, shown among the 4 most frequent responses. None of the respondents reported correctly all the legal rules for generic substitution.

**Table 2:** Legal rules that make generic substitution in pharmacies (*N* = 1237)

Legal rule (LR)	Correct answer Number (%)^a^
The same active substance (LR1)	1004 (81.2)
Physician’s consent (LR2)	711 (57.5)
The same total dose (LR3)	648 (52.4)
The same drug strength (LR4)	526 (42.5)
The same dosage form (LR5)	488 (39.5)
Patient’s consent (LR6)	476 (38.5)
The same route of administration (LR7)	463 (37.4)
“Branded substitution not permitted” is not indicated on the prescription (LR8)	316 (25.5)
Lower patient’s co-pay (LR9)	219 (17.7)

^*a*^ *It exceeds total number (100%) because of possibility of multiple choice*

**Fig. 1:**
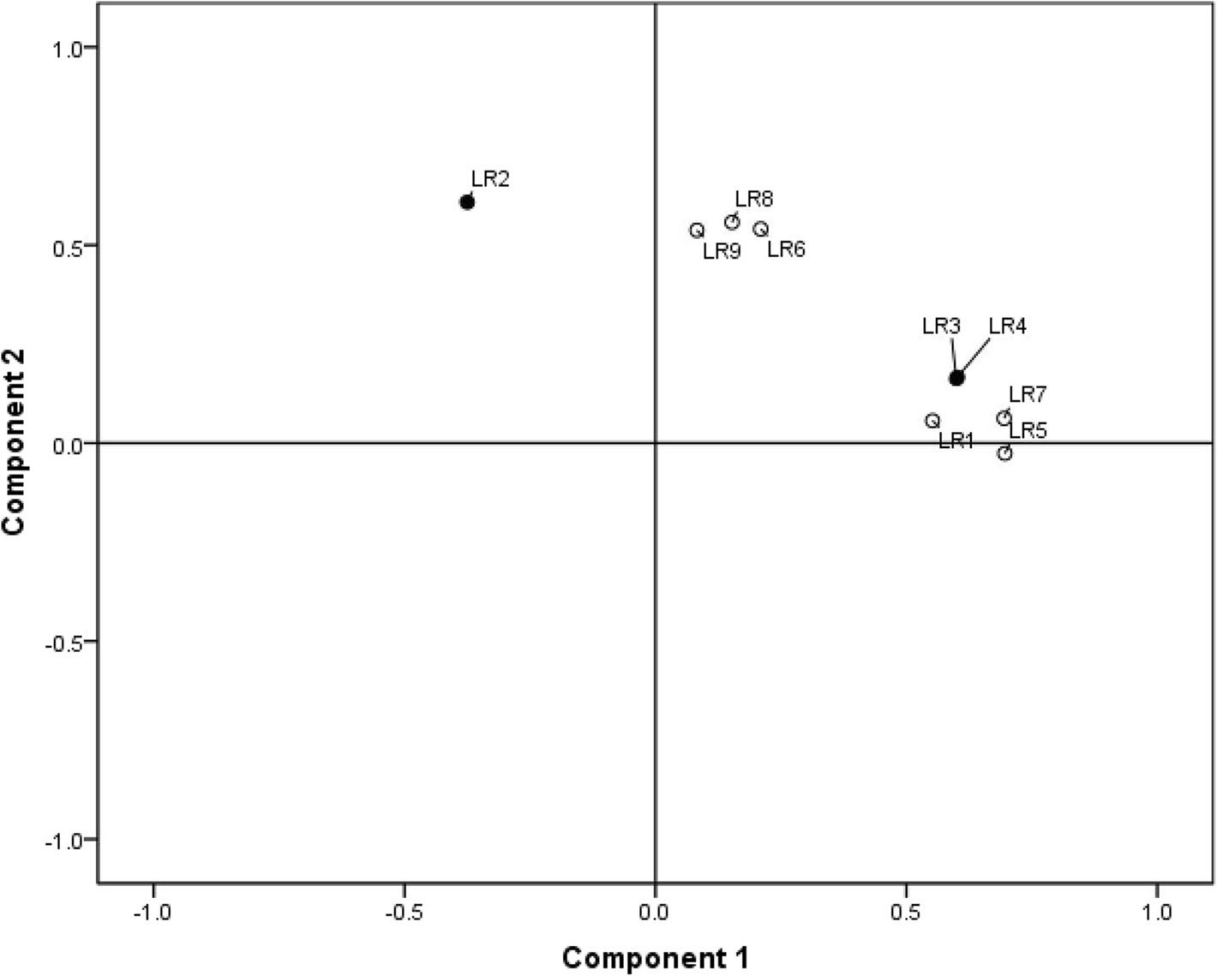
Factor of consensus and factor of agreement in the view of legal rules. Legend: Factor of consensus (LR2 Physician’s consent; LR6 Patient’s consent; LR8 Branded substitution not permitted as indicated on the prescription; LR 9 Lower patient’s co-pay); Factor of agreement (LR1 Same active substance; LR3 Same total dose; LR4 Same drug strength; LR5 Same drug form; LR7 Same route of administration); incorrect answers in bold (LR2, LR4)

Based on the respondents´ answers, factor analysis has identified two factors in all 9 items focused on legal requirements for generic substitution (Fig. 1). The correlation of both factors with individual items has shown that the physicians´ interpretation of legislative requirements is more precise than their actual knowledge. This means that physicians overestimated the law, as they considered some rules valid, even if the law does not require them. The first factor explained 24.5% of the questionnaire variability and mostly correlated with items concerning the need for accordance on generic substitution, i.e. how much the substituted drugs must be equivalent to each other (“factor of accordance”). For an item targeting the rule of strength equivalence, the factor of accordance correlated positively with the incorrect answer and negatively with the correct answer. Respondents with the maximum score in factor of accordance answered this question (incorrectly) positively, thus showed higher compliance with rules of providing generic substitution than it was required by the law. The second factor explained 14.7% of the questionnaire variability and correlated mostly with questions focusing on the necessary consensus among the persons involved in generic substitution (“factor of consensus”). Indeed, for an item targeting the physician’s consent, the factor of consensus correlated negatively with the correct answer. Respondents with the maximum score regarding this factor, considered beyond the law that generic substitution requires physicians´ consent.

The interpretation presented above was supported by reliability analysis. The Cronbach alpha of all 9 questions was 0.318, if all correct and incorrect answers were analyzed. Both rules of strength equivalence and physicians´ consent showed an increase in Cronbach alpha if those items had been deleted (0.528 or 0.336, respectively). The rule of strength equivalence showed a significantly negative item-total correlation (− 0.393), whereas the physician’s consent close to zero (0.038). However, if the attitudes towards these two items were taken into account (i.e., how respondents answered), Cronbach alpha increased to 0.553. Overall, it can be said that the better knowledge the respondents showed, the less they were aware of the consensus.

There were no statistically significant associations between the knowledge of legal rules including both factors and sociodemographic or medical specialty characteristics. The median of the knowledge score among different medical specialties was 4.

### Attitudes towards performing generic substitution in pharmacies

**Fig. 2:**
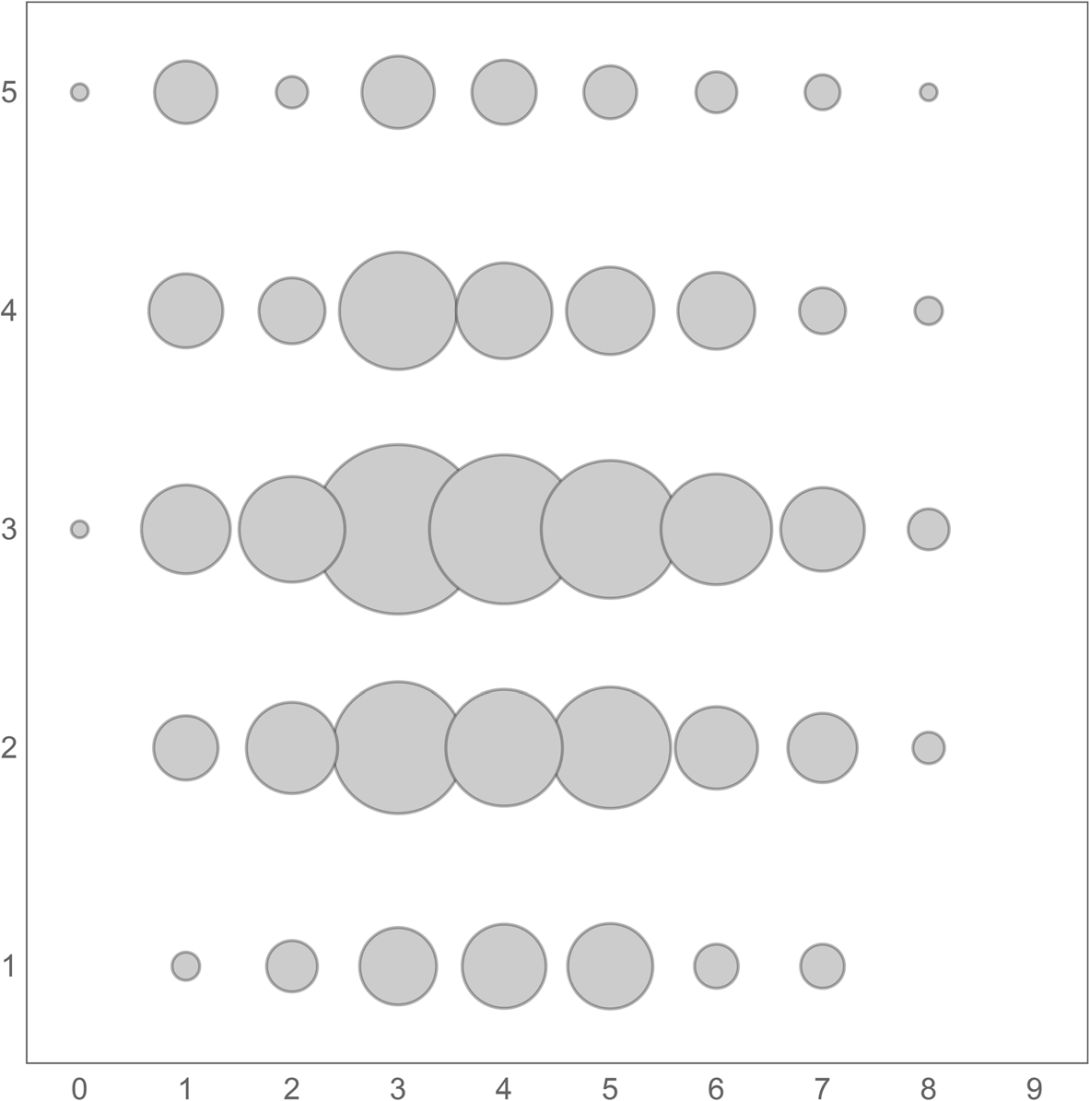
Correlation between attitudes towards and understanding the legislation for generic substitution. Legend: x axis: understanding the legislation for generic substitution (each correct answer scored one point, maximum of nine points); y axis: attitudes towards generic substitution in pharmacy (1 – very positive, 2 – rather positive, 3 – neutral, 4 – rather negative, 5 – very negative)

The majority of physicians (497; 40.2%) felt neutral about performing generic substitution in pharmacies, while positive and negative attitudes were reported by 434 (35.1%) and 306 (24.7%) respondents, respectively. Generic substitution was very negatively considered by 88 (7.1%) physicians. Physicians with completed specialized qualification more often expressed negative attitudes (*p* < 0.01), however with small effect size. A significant correlation between understanding the legislation for generic substitution and attitudes towards generic substitution was revealed (tau = 0.06; *p* < 0.05); the better understanding the legislation, the more positive attitude towards generic substitution (Fig. 2). Respondents with higher knowledge score and higher factor of accordance perceived generic substitution more positively (p < 0.05). On the other hand, higher factor of consensus negatively correlated with the attitudes of providing generic substitution at the pharmacy, but non-significantly.

### Comparison of the knowledge and attitudes between the GP cohorts

The knowledge of legislation in the group of GPs for adults compared with the results obtained in the previous study [19] was lower with a mean score 3.9 versus 4.7 (η^2^ = 0.172; *p* < 0.001 with large effect size). Similarly, both groups significantly differed in their attitudes towards performing generic substitution in pharmacies (η^2^ = 0.172; p < 0.001 with large effect size). Positive and rather positive (7.7 and 28.7%) attitudes were reported by GPs in the current study compared to the previous one (5.3 and 16.0%, respectively). Neutral, rather negative or negative attitudes were currently reported by 42.0, 12.5, and 9.1% GPs, compared to 19.4, 36.1 and 23.2% attitudes reported previously.

## Discussion

The findings of our study suggest that the Czech physicians have rather positive attitudes towards generic drugs and generic substitution, and consider generic drugs therapeutically equivalent and similarly bioequivalent to the brand name drugs, as well as generic drugs themselves to each other. Compared to the previous survey on GPs [19], positive opinions on therapeutic equivalence and bioequivalence were shown to be better and the improvement may be justified, among others, due to growing experience with generic substitution in the CR, more precise knowledge of principles of bioequivalence studies or greater interest in this issue. In recent years for example, relatively extensive relevant studies have also been published that have not refuted the therapeutic equivalence of brand name and generic cardiovascular drugs [26]. Since the opinions on ensuring safety measures and guaranteeing the quality of generic drugs compared to their original counterparts were also very positive, higher confidence in the processes of approval of medicinal products on the Czech market can be expected. Similarly, positive opinions have been reported in studies conducted in developed countries, where transparent, clear, and effective regulatory rules for generic substitution had been set up [27, 28]. On the other hand, healthcare professionals from countries with a less mature healthcare system appeared to have more concerned about the manufacturing sources of generics [20, 29, 30].

From the associations of socio-demographic characteristics, positive opinions on bioequivalence can be highlighted in ambulatory physicians, who are presumably more apt to prescribe drugs with higher benevolence than in physicians in the inpatient setting. The restrictions imposed by the hospital positive lists and the influence of pharmaceutical companies may also lead to lower willingness to prescribe generics [27]. However, the associations of individual results with sociodemographic characteristics should be generally considered with caution and confirmed in future research. Such results differ and occasionally are contradictory in the literature. Therefore, it is not possible to summarize the influence of age, length of practice or specialization on the physicians´ attitudes towards generic substitution. The results can be associated with the diversity of national healthcare policies, including various strategies of regulatory authorities, physicians´ knowledge, or the general development of the given country, as well as with the changes that may occur over time [11, 28].

It is alarming that a relatively large group of respondents did not express any opinion, either on drug equivalence, or on quality and safety guarantees. This fact, accompanied with the lack of confidence in generic substitution, can limit that generic drugs and generic substitution be fully adopted by physicians [11]. With regard to ADRs, no opinion was expressed by younger practitioners in specialized training, which may be explained by their low awareness or inexperience with providing generic substitution in clinical practice [15]. In older physicians, skepticism was more prevalent as they viewed generics are less safe. However, the majority of respondents have not witnessed recently any ADRs or drug-related problem. In case of any occurrence, allergic reactions were predominant, which is in line with previous research results [19]. Some drugs (e.g. antiepileptics) can be signaled to increase the risk of ADRs or lack of efficacy during generic substitution in a particular patient [31], however, data from large randomized controlled trials are either missing or the findings of the observational studies do not confirm their conclusions. Despite this fact, the substitution cannot be performed in all patients and all drugs [32]. It is also possible that our respondents did not have such frequent contact with these groups of drugs or patients or they failed to identify any ADRs.

More than half of the respondents reported that the primary principle of generic substitution is based on reducing the healthcare cost for patients, which may be debatable. As pharmaceutical companies compete for their position on the market, the prices (including the need for patient co-payment) rapidly decrease after the original patent expired. Therefore, generic drug prices may remain more or less equal, while brand name drugs tend to stay closer to the generic price [5]. In some countries, the reimbursement of generic and brand name drugs is the same, and the financial relief for the patient is therefore not a generic substitution motivating priority [33]. Consequently, the choice of drug to be prescribed by the physician may be affected by the specific brand to which the physician wishes to be loyal; also marketing efforts by the pharmaceutical industry can play a role [28]. The consumer (i.e., the patient) does not even have to see the difference in the price and may choose the drug according to their own preferences. Therefore, it is necessary to underline the importance of cooperation between patients and physicians regarding the right choice of a suitable therapy, as well as the relationship with pharmacists, who should consider all the risks of medication (e.g. contraindications, ADRs, drug interactions) and patients characteristics, in particular if any change has occurred in the pharmacotherapy. Inappropriate generic substitution may lead to the patient’s medication non-adherence and early discontinuation of the therapy, thereby losing confidence in healthcare professionals [34]. In the other way round, sufficient understanding of the patient’s treatment plan result in the patients´ increased care for their own health, hence to better adherence [35, 36]. Moreover, regulatory authorities and professional societies provide healthcare professionals with guidelines or lists of drugs unsuitable for generic substitution, especially drugs with a narrow therapeutic window [20]. Compliance with these principles is important for safe medication practices, as well and similarly, higher awareness among healthcare professionals towards generic substitution is perceived as a positive drug policy tool, and never as a possible means to cause harm to the patient [37].

Controversies or negative attitudes often result from poor knowledge of the principles of generic substitution. As mentioned above, the understanding of therapeutic equivalence and bioequivalence seems to have improved among Czech physicians. The appropriate knowledge of generic substitution legal rules is still contradictory. None of the participants responded correctly to all the questions on knowledge of legislation, however, they prompted stricter rules for generic substitution in the pharmacy (physician’s consent and same strength), although both of those rules can be circumvented by avoiding generic substitution by indicating” dispense as written” on the prescription, or dispensing different strength in a modified dosing, respectively. Similar patterns can be seen in other studies. For example, up to 65% of physicians denied providing a liberty to the pharmacists for changing brand name drug for generic medicine in Malaysia or Pakistan [29, 38].

In the CR, therefore, patients have the right to decide themselves if the proposed generic substitution is convenient, even if it is required that they understand the generic substitution principles, as explained by the pharmacist. According to the published literature, pharmacists have shown a deeper knowledge and more positive attitudes towards generic substitution than the prescribing physicians, which may be due to their former education focused more on drugs including generic substitution [3, 20]. The pharmacists performing generic substitution in the CR play an important role in the partnership with the patients, both aiming to reveal the risk factors of generic substitution for the patients, as well as to identify the drugs and their indications unsuitable for generic substitution. Interestingly, whereas the knowledge of GPs showed decreased scores, compared to the previous survey [19], the attitude towards providing generic substitution in pharmacies has improved. Lower knowledge can be acknowledged to the selection of respondents in the previous study (conference participants, i.e. probably more educated participants); on the other hand, the improvement of attitudes can be explained by growing experience with generic substitution, as well as improved interdisciplinary cooperation. Providing information on generic substitution by pharmacists to patients demonstrated to have a major impact on the perceptions of physicians in a study by Lewek [39].

### Strengths and limitations

This study provides results of a representative sample survey on the physicians´ attitudes towards generic drugs and generic substitution in the CR with a high response rate, which is rather unique, especially compared to similar studies published so far. We can consider our study to be novel as reflects the current perception of generic drugs and generic substitution by the prescribing physicians. Even though this was a questionnaire survey, questions were asked face-to-face, respondents were not honored, the scope of the questionnaire was not too large, as well as the clarity of questions was piloted and published a forehand [19]. On the other hand, it is still a cross-sectional study, which does not evaluate the situation over time, and had no aim to identify variable factors affecting the attitudes of physicians. The latter could be facilitated by qualitative research with open-ended questions in the future to understand the issues concerning generic drugs and generic substitution more in detail, although this may gain smaller group of respondents [12, 40].

## Conclusion

The study showed insufficient awareness of generic drugs and generic substitution among Czech physicians. Higher knowledge of Czech legislation seemed to improve their perception of providing generic substitution in pharmacies, however, they frequently overestimated legislative requirements. Attitudes towards generic substitution are slightly more positive compared to opinions observed at the time of introducing generic substitution into the market in the CR, however, quite large proportion of physicians was not able to express any opinion in terms of quality, effectiveness, or regulatory standards. Majority physicians have experienced no drug-related problem; still, a better understanding of generic substitution by physicians can contribute to higher patient safety during pharmacotherapy.

## Supplementary information

**Additional file 1.** Questionnaire survey. Specific items included in the questionnaire concerning statements related to brand name drugs, generic drugs and generic substitution, previous experience with drug-related problems of generic drugs and generic substitution, understanding of legal rules for generic substitution in the Czech Republic, and attitudes towards performing generic substitution in pharmacies.

## Data Availability

The datasets used and analyzed during the current study are available from the corresponding author on reasonable request.

## Abbreviations

ADR: Adverse Drug Reaction
AUC: Area under Curve
C_max_: Maximum Plasma Concentration
CR: Czech Republic
GP: General Practitioner
IQR: Inter Quartile Range
SD: Standard Deviation
