# Supplementary material for: Representative sample survey on factors determining the Czech physicians’ awareness of generic drugs and substitution
Source: BMC Health Serv Res. 2019 Oct 30;19:777. doi: 10.1186/s12913-019-4631-y (PMC6822393; doi:10.1186/s12913-019-4631-y)
Supplement: Supplementary file 1 — Additional file 1. Questionnaire survey. Specific items included in the questionnaire concerning statements related to brand name drugs, generic drugs and generic substitution, previous experience with drug-related problems of generic drugs and generic substitution, understanding of legal rules for generic substitution in the Czech Republic, and attitudes towards performing generic substitution in pharmacies. [file 12913_2019_4631_MOESM1_ESM.docx]

**Please, express your opinions on statements related to brand name drugs and generic drugs and generic substitution** (1=strongly agree; 2=agree; 3=neutral; 4=disagree; 5=strongly disagree).

- Every generic drug is therapeutically equivalent to the brand name drug.
- Every generic drug is therapeutically equivalent to any other generic drug.
- I need more information on results of bioequivalence studies to make a responsible decision on the use of generic drugs.
- Every generic drug is bioequivalent to the respective brand name drug.
- Every generic drug is of lower quality than the brand name drug.
- Every generic drug is less effective than the brand name drug.
- Every generic drug cause more adverse drug reactions than the brand name drug.
- Every generic drug is less costly than the brand name drug.
- The law imposes the same safety requirements on both generic and brand name drugs.
- Generic substitution reduces drug costs in the patient´s pharmacotherapy.

**What are your attitudes towards performing generic substitution in pharmacies?**

(1=positive; 2=rather positive; 3=neutral; 4=rather negative; 5=negative)

**Choose the conditions that make generic substitution in pharmacies in the Czech Republic.**

1. The same active substance.
2. The same total dose.
3. Patient´s consent.
4. The same route of administration.
5. The same dosage form.
6. Physician´s consent.
7. The same drug strength.
8. Lower patient’s co-pay.
9. “Branded substitution not permitted” is not indicated on the prescription.

**Have you encountered any problem with your patients in relation to generic substitution or generic drugs, such as an increased incidence of adverse effects or duplication, in the last 3 months?**

1. No
2. yes, please refer which: ……………………………
